# Golden plaster for pain therapy in patients with knee osteoarthritis: study protocol for a multicenter randomized, double-blind, placebo-controlled trial

**DOI:** 10.1186/1745-6215-14-383

**Published:** 2013-11-13

**Authors:** Jin-Tao Liu, De-Zhi Tang, Xiao-Feng Li, Zhi-Gang Zhang, Wan-Bo Ji, Shuai Tao, Yong-Jun Wang, Hong Jiang

**Affiliations:** 1 Suzhou Hospital of Traditional Chinese Medicine, 889 Wuzhongxi Road, Suzhou, Jiangsu 215009, People's Republic of China; 2 Longhua Hospital, Shanghai University of Traditional Chinese Medicine, 725 Wan-Ping South Road, Shanghai 200032, PR China; 3 Zhangjiagang Hospital of Traditional Chinese Medicine, Zhangjiagang, Jiangsu 215600, People's Republic of China; 4 Taizhou Hospital of Traditional Chinese Medicine, Taizhou, Jiangsu 222500, People's Republic of China

**Keywords:** Clinical trials, Golden plaster, Knee osteoarthritis, Visual analog scale

## Abstract

**Background:**

Osteoarthritis is a relatively common musculoskeletal disorder that increases in prevalence with age. Worldwide, knee osteoarthritis is one of the leading causes of disability, particularly in the elderly. In numerous trials of agents for long-term pain therapy, no well-established and replicable results have been achieved. Complementary and alternative medical approaches have been employed for thousands of years to relieve knee osteoarthritis pain. Among herbal medicines, the golden plaster is the preferred and most commonlyused method in China to reduce pain in patients with knee osteoarthritis, as it causes few adverse effects. The purpose of this study will be to evaluate the efficacy and safety of golden plaster on pain in patients with knee osteoarthritis.

**Methods/Design:**

This study will be a multicenter randomized, double-blind, placebo-controlled trial. A total of 320 participants aged 45 to 79 years with knee osteoarthritis, whose scores on a visual analog scale (VAS) are more than 20 mm,will be randomly allocated into a treatment group and a control group. A golden plaster will be administered externally to participants in the treatment group for 2 weeks, while the control group will receive a placebo plaster externally for 2 weeks. Follow-up will be at regular intervals during a 4-week period with a VAS score for pain, quality of life, and complications.

**Discussion:**

This study will be a methodologically sound randomized controlled trial to assess pain relief after the intervention of golden plaster, compared to a placebo intervention in patients with knee osteoarthritis.

**Trial registration:**

ClinicalTrials.gov identifier: ChiCTR-TRC-13003418

## Background

Osteoarthritis (OA), a progressive joint disorder, is a common health issue in the aging population worldwide [1,2]. The prevalence rate of OA is 28% in people over 45 years old and 37% in people over 65 years old [3]; thus, its prevalence and health impact increase with age. Knee OAis one of the leading causes of disability, particularly in the elderly. The aim of OA treatmentisto reduce or control pain, improve physical function, prevent disability, and enhance quality of life. The non-surgical management of OA, namely pharmacologic treatment combined with physical therapy, is utilized more and more widely [4,5]. Pharmacologic treatments include oral analgesics, acetaminophen, ibuprofen, and traditional Chinese medicinal (TCM) products [6,7]. Several systematic reviews have outlined and showed the effectiveness of muscle strengthening or aerobic exercises in the osteoarthritic population [8-11]. Acupuncture and Tuina, two kinds of the physical therapy, have also been used for the management of OA [6,7]. Despite numerous trials, investigators have not been able to establish replicable relief of long-term pain.

Complementary and alternative medicine has been employed over thousands of years to relieve knee OA pain. Plasters are a very important therapeutic method in TCM, inducing muscle relaxation, invigorating blood circulation, and improving electrolyte balance and bile release [12,13]. Among herbal medicines, the golden plaster is the preferred and most commonly used method in China for pain reduction in patients with knee OA, as they cause very few adverse effects. Due to the absence of a gold standard for the treatment of knee OA, this trial will be conducted to detect the effectiveness of these herbal medicines compared to a placebo-controlled group. Therefore, we designed a randomized, double-blind, placebo-controlled trial of golden plaster in patients with knee OA.

## Methods/Design

### Study design

This clinical trial will be a multicenter, randomized, double-blind, placebo-controlled trial. Subjects will be enrolled at four hospitals: i) Suzhou Hospital of TCM; ii) Longhua Hospital affiliated to Shanghai University of TCM; iii) Zhangjiagang Hospital of TCM; and iv) Taizhou Hospital of TCM.

The Ethics Boards of the Suzhou Hospital of TCM, Longhua Hospital affiliated to Shanghai University of TCM, Zhangjiagang Hospital of TCM, and Taizhou Hospital of TCM have approved this study. Each participating center has obtained local Institutional Review Board approval. All study participants will sign the written informed consent before participation.

### Inclusion criteria

The inclusion criteria are as follows: age 45 to 79 years; symptomatic knee OA with a pain of at least 20 mm on a 100-mm visual analog scale (VAS); American College of Rheumatology criteria for symptomatic knee OA assessed by a rheumatologist [14]; ability to read, speak, and understand English or Chinese; and capability of understanding the study requirements and willingness to cooperate with the study instructions.

### Exclusion criteria

Exclusion criteria are as follows: psoriatic arthritis; lupus or cancer; severe cardiac or renal impairment; significant trauma to knees, including arthroscopy or significant injury to ligaments or the menisci of the knee within one year preceding the study; allergy to plaster.

### Recruitment

Participants will be recruited through advertisements in local newspapers, bulletin boards, and on the websites of local medical centers. All patients will be screened initially by baseline assessment with regard to selection criteria before randomization. If inclusion criteria are met and the informed consent form is signed, the patient will be sent to randomization.

### Randomization

The teletherapist, a research nurse, will then register the participant into the database for randomization. If the participant is ready to be randomized, the teletherapist enters “yes”; the site-specific randomization program behind the form displays the participant’s group assignment number (placebo versus golden plaster). Site-specific randomization lists will be computer-generated (i.e., generated by an individualized basic visual code program) and concealed from the researchers by a senior data manager who is not involved in the study. This information will remain confidential and is not shared with the study sites, in accordance with the CONSORT guidelines. This trial uses a prospective, randomized, outcome-blinded design, in which all outcome assessments are made by a research assistant blinded to treatment allocation and uninvolved in patient consent and management.

### Intervention

**Figure 1:**
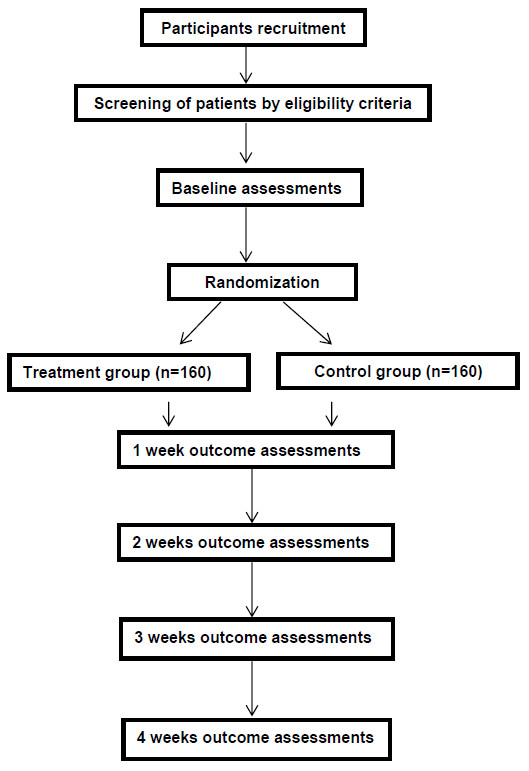
Study flow diagram, including recruitment, eligibility, screening, randomization, and outcome assessments.

This study will be conducted in accordance with the requirements outlined in the Declaration of Helsinki, and approved by the appropriate Institutional Review Boards. Each participant will sign the written informed consent form before undergoing any examination or study procedure, in compliance with Good Clinical Practice. Eligible patients will be randomized into one of the two groups: placebo and golden plaster. All drugs will be administered externally for 2 weeks. Participants are given in-person instructions for using the plaster by our research nurses, who will be trained before this study. The trial will be double-blind: the treating physicians, subjects, investigators and statisticians are unaware of treatment assignment. Randomization of subjects will occur centrally using a random number generator. Patientvisitswill be performed at base line, and 1-, 2-, 3-, and 4-week follow-up after treatment (Figure 1). Assessments will be made during all five visits. Used plasters will be reclaimed at the 1- or 2-week follow-up visit.

The investigational group will receive one medicated plaster, to be used twice a day (morning and evening, approximately every 12 hours and at the same time each day) for 14 consecutive days. The golden plaster is a 10 × 15 cm medicated adhesive patch, composed of Trichosanthes root, turmeric, angelica, Atractylodes, licorice, rhubarb, Phellodendron, Arisaema, Magnolia bark, tangerine peel, sesame oil, Huang Dan, and a hydrophilic adhesive vehicle.

The control group will receive placebo plasters (containing the hydrophilic adhesive vehicle only), which are identical to the tested formulation in terms of texture, size, color, and odor. The tested products will be packed and labeled, according to current Good Manufacturing Practice and legal requirements, by the Suzhou Hospital of TCM to ensure the participants and all personnel involved in the study will remain fully blinded as to the identity of the treatment administered. During the process, patients will not be allowed to use other drugs. If they do so, patients are required to provide feedback to the investigators and discontinue involvement in the study.

### Outcome measures

#### Primary outcome measure

The primary efficacy endpoint of the study will be the VAS, which is a pain score ranging from 0 mm (no pain) to 100 mm (worst pain ever experienced) [15]. Operationally, the VAS score is usually a horizontal line, 100 mm in length, anchored by word descriptors at each end. Patients mark the point on the line of current pain. The VAS score is then determined by measuring in millimeters from the left hand end of the line to the point that the patient marked. The VAS score will be measured during all the assessment visits (baseline and 1-, 2-, 3-, and 4-week follow-up).

#### Secondary outcome measure

Health related quality of life will be measured using the SF-36 questionnaire [16]. The SF-36 questionnaire is a widely used measure of general health status, consisting of eight parts: physical functioning, role-physical, bodily pain, general health, vitality, social functioning, role-emotional, and mental health.

### Safety assessments

All subjects are to be questioned about adverse events during the treatment at each visit, and all adverse events reported will be analyzed, regardless of the investigators’ assessments of causality. Safety will be assessed by complete blood cell count, erythrocyte sedimentation rate, blood chemistry, and urinalysis.

### Sample size

We will estimate the required sample size for this trial based on the following calculation: n = 2σ^2^ × f(α, β)/(μ1-μ2)^2^[17]. First, we estimate that an absolute improvement of 4.7 (from μ1 to μ2) in VAS score is likely the smallest clinically-relevant difference for patients with OA. Second, we assume that standard deviation of VAS score may be 2.9 (σ = 2.9) at baseline [18]. Based on these assumptions, we will require 84 patients in each group to have at least a 90% power (β = 0.1) and to rule out a two-sided type I error of 5% (α = 0.05). The number of patients actually provides less than 80% power, assuming a withdrawal rate of 20%. Therefore, we will recruit a total of 320 patients, 160 patients in each group.

### Statistical analysis

The data will be collected and analyzed according to the intention-to-treat principle. Standard statistical techniques will be used to describe characteristics of patients in both groups. We will compare baseline characteristics in both groups and, if incomparability appears, we will perform a secondary analysis, adjusting for differences. The primary outcome will be compared between both groups using analysis of variance for repeated measures. All statistical analyses will be performed using SAS version 9.1 or a later version. All statistical tests will be two-sided and the level of significance will be set at 0.05. The last-observation-carried-forward method will be used to input data with dropouts. Continuous variables will be expressed as mean ± SD, and categorical variables as number and percentage [19]. If adjustment for possible baseline incomparability is needed, analysis of covariance will be performed [20].

## Discussion

Despite considerable study, there is no generally effective treatment for patients who suffer from OA. In some contexts, various complementary and alternative medical treatments have been administered for OA in clinical practices [8,10,21]. Some studies have reported the effectiveness of herbal medicine, which is the most popular form of complementary and alternative medical therapy for the treatment of OA, either used alone or concomitantly with usual care [6,22-26]. The quality and small sample sizes of the few trials that have been conducted have made it difficult to reach firm conclusions about these treatments. Well-designed randomized controlled trials are needed to examine the efficacy of TCM treatments for OA. The biggest advantage of the present trial is an external placebo control. The purpose of this study will be to evaluate the basic clinical efficacy and safety data for the golden plaster in the treatment of patients with knee OA.

### Trial status

Patient recruitment for the trial will start from the end of 2013. Data collection will finish at the end of 2015.

## Abbreviations

HRQoL: Health-related quality of life; OA: Osteoarthritis; TCM: Traditional Chinese medicine; VAS: Visual analogue scale.

## Competing interests

The authors declare that they have no competing interests.

## Authors’ contributions

JTL, DZT, XFL, ZGZ, WBJ, ST, YJW, and HJ contributed to the design of this study. JTL, DZT, XFL, ZGZ, WBJ, ST, YJW, and HJ contributed to the creation of the Manual of Procedures, implementation of the study protocol and acquisition of data. JTL, DZT, and XFL drafted the manuscript, and JDH modified the manuscript. All authors provided critical revision and approved the final manuscript.
